# A case of tuberculous endophthalmitis successfully treated with vitrectomy followed by antituberculous agents

**DOI:** 10.1186/s12348-015-0046-z

**Published:** 2015-05-08

**Authors:** Keitaro Hase, Kenichi Namba, Wataru Saito, Shigeaki Ohno, Susumu Ishida

**Affiliations:** Department of Ophthalmology, Hokkaido University Graduate School of Medicine, N15-W7, Kita-ku Sapporo, 060-8638 Japan; Department of Ocular Circulation and Metabolism, Hokkaido University Graduate School of Medicine, Sapporo, 060-8638 Japan

**Keywords:** Tuberculosis, Uveitis, Tuberculous endophthalmitis, Pars plana vitrectomy, Antituberculous agents, *Mycobacterium tuberculosis*, Prednisolone

## Abstract

**Background:**

Tuberculous endophthalmitis is very rare with only 18 reports published worldwide and only a few cases in Japan. We report a case of tuberculous endophthalmitis successfully treated with vitrectomy followed by antituberculous agents.

**Findings:**

An 81-year-old man was referred to us due to the exacerbation of vitreous opacity on his left eye(OS) after he had received the corticosteroid therapy. His best corrected visual acuity was light perception OS, and he had severe intraocular inflammation with fibrin formation in the anterior chamber and dense vitreous opacity. A chest CT showed miliary nodules indicating miliary tuberculosis, and pars plana vitrectomy was performed. Intraoperative observation showed that the vitreous cavity was filled by fibrin, and large elevated subretinal yellow-white lesions were present at the mid-periphery. The patient immediately received triple antituberculous agents orally, and *Mycobacterium tuberculosis* was detected in vitreous fluids. The intraocular inflammation gradually decreased, and the subretinal mass regressed within 2 weeks.

**Conclusions:**

We encountered a case of tuberculous endophthalmitis successfully treated with vitrectomy followed by antituberculous agents. If endophthalmitis is suspected in a patient with systemic tuberculosis infection, prompt vitrectomy along with the administration of antituberculous agents may be necessary.

## Findings

### Introduction

Tuberculosis is a serious infectious disease that has re-emerged as a public health problem in recent years and is ranked as an important cause of death worldwide [1]. Intraocular tuberculosis represents an extrapulmonary form of this disease. Approximately 1% to 2% of patients with tuberculosis show ocular involvement, in the lids, conjunctiva, cornea, sclera, uveal tract, optic nerve, or orbit [2]. The most common form of ocular involvement is uveitis. In tubercular uveitis, posterior or panuveitis is the most common type and includes choroidal tubercules, choroidal tuberculoma, subretinal abscess, and serpiginous-like choroiditis [3]. However, the most frequent type in Japan is retinal vasculitis, while the other types are rare. Moreover, the development from ocular tuberculosis to endophthalmitis is very rare, and only a few cases have been reported in Japan [4]. To the best of our knowledge, only 18 reports have been previously published worldwide, and almost all of these cases underwent enucleation or evisceration [1,5,6] because they rapidly progressed with destruction of the intraocular tissues. We report a case of tuberculous endophthalmitis that resulted in the remission of ocular inflammation by pars plana vitrectomy (PPV) combined with subsequent administration of antituberculous agents.

### Case report

The case was an 81-year-old Japanese man who had visited an ophthalmology clinic complaining of blurred vision of the left eye with periocular pain and was diagnosed as uveitis on his left eye(OS). He had also suffered from left temporal headaches for 2 weeks, and he had undergone corticosteroid pulse therapy based on a suspected diagnosis of temporal arteritis. Two weeks later, the vitreous opacity OS exacerbated, and he was referred to us. He had no systemic diseases except well-controlled systemic hypertension and hyperlipidemia.

Best corrected visual acuity (BCVA) was 1.0 on his right eye(OD) and light perception OS. The right eye showed a normal appearance. In the left eye, slit lamp examination showed pigmented keratic precipitates, Descemet’s membrane folds, and severe cells and flare with fibrin in the anterior chamber. The fundus was not visible due to the dense vitreous opacity. B-mode ultrasonography showed diffuse high density in the vitreous cavity and dome-shaped high-density lesions elevated toward the inside at the site of the temporal peripheral choroid. Single-flash electroretinography (ERG) results were normal OD and non-recordable OS. Examination of serum was normal, including blood counts and liver function, except for an increase in C-reactive protein (1.65 mg/dl). In addition, we were informed that chest computed tomography showed diffuse miliary nodules in both lungs indicating miliary tuberculosis.

**Figure 1:**
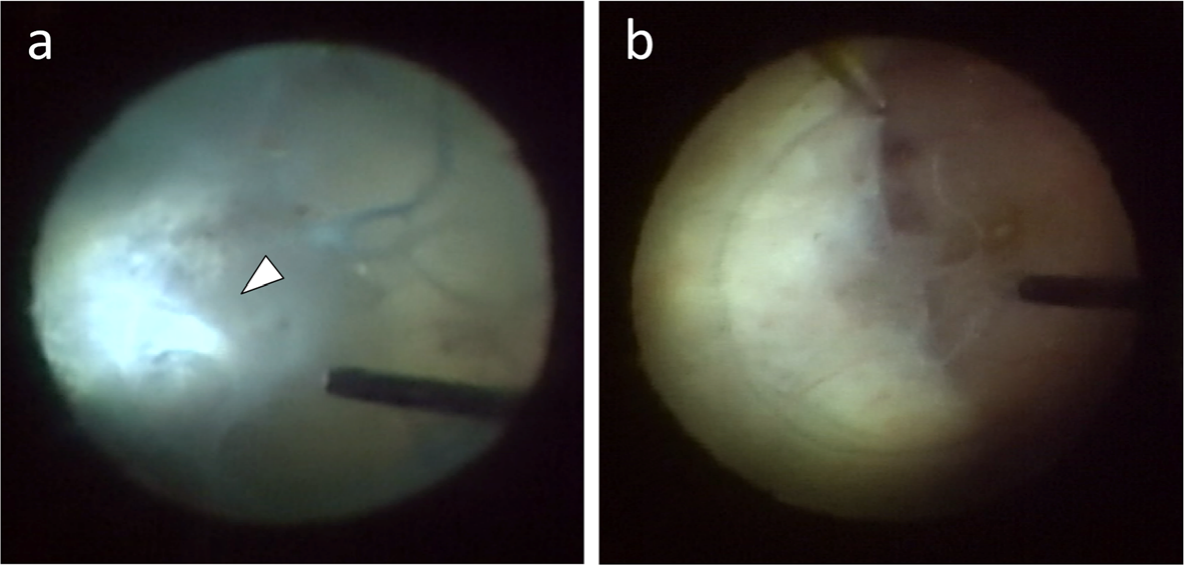
Intraoperative views during PPV. **(a)** Intraoperative view in PPV showing numerous fibrins (arrow) attached above elevated lesions at the temporal mid-periphery. **(b)** Intraoperative view in PPV showing multiple large elevated dome-shaped subretinal yellow-white lesions at the temporal to inferior mid-peripheral area.

Since these findings suggested infectious endophthalmitis, the patient underwent PPV immediately. Intraoperatively, the vitreous cavity was filled by dense opacity with numerous fibrins (Figure 1a). Multiple large, elevated, dome-shaped subretinal yellow-white lesions fused to each other were present at the temporal to inferior mid-periphery (Figure 1b). No retinal break or retinal detachment was detected. Vitreous gel with fibrin was removed as much as possible with intravitreal irrigation with balanced salt solution containing ceftazidime and vancomycin. After the operation, the patient immediately received triple antituberculous agents orally (isoniazid 300 mg/day, ethambutol 750 mg/day, and rifampin 450 mg/day). Later, *Mycobacterium tuberculosis* was detected by bacterial culture from the gastric juices and vitreous fluids. From these results, the patient was diagnosed with tuberculous endophthalmitis.

**Figure 2:**
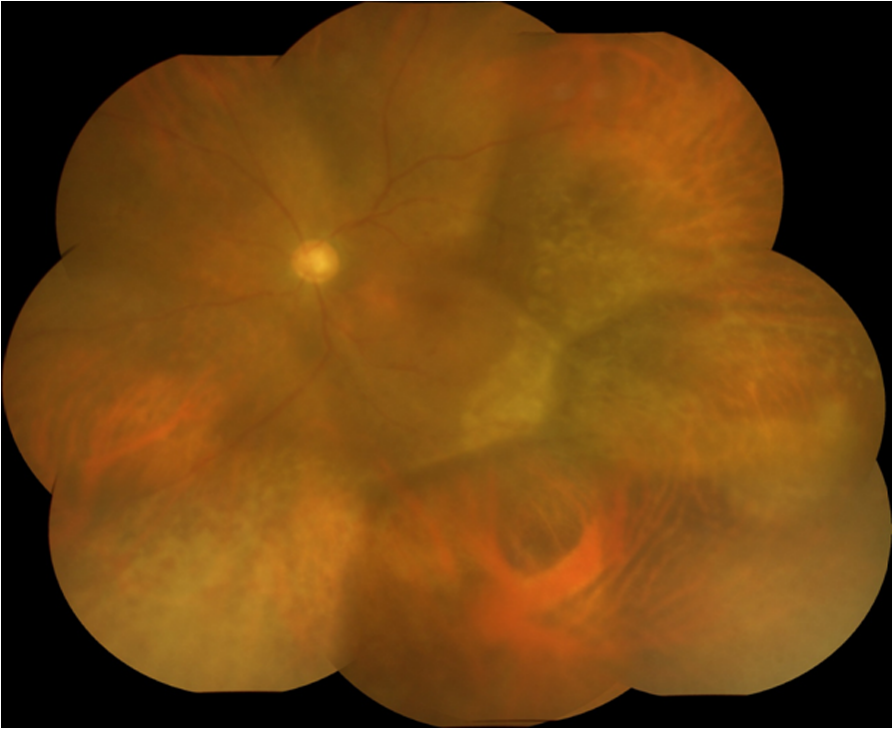
Fundus photograph after PPV. Fundus photograph of the left eye 2 weeks after PPV. Elevated subretinal yellow-white lesions have already disappeared, with retinal atrophy remaining at that site.

Two weeks after the operation, the anterior chamber inflammation decreased to 1+ flare and 2+ cells without any vitreous opacity. Elevated subretinal yellow-white lesions regressed with remaining slight retinal degeneration at the site corresponding to the area where subretinal yellow-white lesions had previously existed (Figure 2). BCVA improved to 0.05 OS. Goldmann perimetry revealed inferotemporal island scotoma with central scotoma. ERG results continued to be non-recordable OS.

Two months after the operation, the patient showed re-exacerbation of intraocular inflammation OS despite continuing antituberculous agents. BCVA decreased to light perception OS. Anterior chamber inflammation increased to 2+ flare and 4+ cells. The fundus could not be visualized due to diffuse vitreous opacity with fibrin formation in the anterior vitreous. Although systemic antibacterial and antifungal drugs were added, intense intraocular inflammation continued. However, when oral prednisolone 20 mg/day was initiated, inflammation in the anterior chamber and the vitreous resolved dramatically, and BCVA improved to 0.15. The oral prednisolone was gradually decreased and was stopped 11 months after the operation. The antituberculous agents were also stopped 13 months after the operation. No recurrence of intraocular inflammation was seen, and the final visual acuity was 0.1 OS.

### Discussion

We encountered this case of tuberculous endophthalmitis diagnosed by systemic screening and bacterial culture of vitreous fluids. In the early stage after the operation, we initiated antituberculous agents, which led to the remission of endophthalmitis and BCVA improvement.

According to previous reports, the visual prognosis for tuberculous endophthalmitis is very poor. Of the 13 cases of this disease reported before 2000, 12 eventually underwent enucleation or evisceration while 1 case showed spontaneous remission [1,5]. From 2001 onward, three out of five cases underwent enucleation while endophthalmitis regressed after PPV in the remaining two cases [1,6]. These observations suggest that the visual prognosis for this disease is poor despite the progression of vitrectomy techniques. This disease often occurs in developing countries that have high-indigenous rates of tuberculosis. Patients with tubercular uveitis are likely to be diagnosed with idiopathic uveitis and are treated solely with local and/or systemic corticosteroids [7]. In the present case, we speculate that the systemic corticosteroid therapy that had been performed at another hospital caused the progression from choroidal tuberculoma to endophthalmitis. Investigations of tuberculosis for the eyes and systemic tissues might be also insufficient. Moreover, new diagnostic tools, such as polymerase chain reaction of *M. tuberculosis* using aqueous or vitreous fluids, also have low sensitivity [7]. Therefore, ophthalmologists should suspect tuberculosis routinely as the cause of endophthalmitis and should perform systemic screening for tuberculosis and analysis of ocular fluids if necessary. In such cases, the decision of whether to start empirical antituberculosis treatment in the absence of positive results is difficult. More sensitive and rapid tests for ocular tuberculosis are urgently needed to guide such cases.

In our case, the recurrence of endophthalmitis occurred 2 months after PPV. Inflammation dramatically resolved with systemic prednisolone therapy, suggesting that the pathological condition was due to an allergic reaction to M. *tuberculosis* components and not due to the recurrence of tubercular infection. This reaction may be very similar to the Jarisch-Herxheimer reaction, which is an exacerbated inflammation seen in patients with tuberculosis just after starting systemic antituberculous treatment. Therefore, when inflammation recurs or worsens in patients with ocular tuberculosis, ophthalmologists should consider the possibility of an allergic reaction to the bacterial components.

### Conclusions

In conclusion, we encountered a case of tuberculous endophthalmitis diagnosed by analysis of ocular fluids obtained by PPV. Triple antituberculous oral agents were initiated from the early stage after the operation, and endophthalmitis dramatically disappeared. If endophthalmitis is suspected in the patient with systemic tuberculosis infection, prompt vitrectomy along with the administration of antituberculous agents may be necessary.

## Consent

Written informed consent was obtained from the patient.

## Abbreviations

BCVA: best corrected visual acuity
CT: computed tomography
ERG: electroretinography
PPV: pars plana vitrectomy
